# Supporting Educational Needs of Older Adult Learners: Strategies for Virtual Transitioning and Student Engagement

**DOI:** 10.1093/geroni/igab046.1496

**Published:** 2021-12-17

**Authors:** Katherina Terhune, Sara Conwell, Amy Danzo, Allyson Graf, Suk-Hee Kim

**Affiliations:** 1 Northern Kentucky University, Lexington, Kentucky, United States; 2 Northern Kentucky University, Highland Heights, Kentucky, United States

## Abstract

The pandemic has revealed a multitude of challenges disproportionately impacting older adults, including older adult learners. Institutions of higher education are uniquely positioned to respond to various challenges using the guiding framework of the Age-Friendly University global initiative. This presentation highlights how preexisting university student support practices and services were adapted to provide older adult learners with guidance for navigating their educational needs during the pandemic. Specifically, it expands on strategies utilized by Adult Learner Programs and Services to effectively pivot to virtual services to support the advising and programming needs of older adult learners. Survey data identifying areas of interest for virtual programming for older adult students will be explored. Recommendations will be discussed for promoting effective transitioning to virtual support systems, preserving student engagement and intergenerational learning, and advocating for aging to remain central to university diversity and inclusion initiatives.

